# AutozygosityMapper: Identification of disease-mutations in consanguineous families

**DOI:** 10.1093/nar/gkac280

**Published:** 2022-04-30

**Authors:** Robin Steinhaus, Felix Boschann, Melanie Vogel, Björn Fischer-Zirnsak, Dominik Seelow

**Affiliations:** Exploratory Diagnostic Sciences, Berliner Institut für Gesundheitsforschung, Berlin 10117, Germany; Institut für Medizinische Genetik und Humangenetik, Charité – Universitätsmedizin Berlin, corporate member of Freie Universität Berlin and Humboldt-Universität zu Berlin, Berlin 13353, Germany; Institut für Medizinische Genetik und Humangenetik, Charité – Universitätsmedizin Berlin, corporate member of Freie Universität Berlin and Humboldt-Universität zu Berlin, Berlin 13353, Germany; Exploratory Diagnostic Sciences, Berliner Institut für Gesundheitsforschung, Berlin 10117, Germany; FB Mathematik und Informatik, Freie Universität Berlin, Berlin 14195, Germany; Institut für Medizinische Genetik und Humangenetik, Charité – Universitätsmedizin Berlin, corporate member of Freie Universität Berlin and Humboldt-Universität zu Berlin, Berlin 13353, Germany; Exploratory Diagnostic Sciences, Berliner Institut für Gesundheitsforschung, Berlin 10117, Germany; Institut für Medizinische Genetik und Humangenetik, Charité – Universitätsmedizin Berlin, corporate member of Freie Universität Berlin and Humboldt-Universität zu Berlin, Berlin 13353, Germany

## Abstract

With the shift from SNP arrays to high-throughput sequencing, most researchers studying diseases in consanguineous families do not rely on linkage analysis any longer, but simply search for deleterious variants which are homozygous in all patients. AutozygosityMapper allows the fast and convenient identification of disease mutations in patients from consanguineous pedigrees by focussing on homozygous segments shared by all patients. Users can upload multi-sample VCF files, including WGS data, without any pre-processing. Genome-wide runs of homozygosity and the underlying genotypes are presented in graphical interfaces. AutozygosityMapper extends the functions of its predecessor, HomozygosityMapper, to the search for autozygous regions, in which all patients share the same homozygous genotype. We provide export of VCF files containing only the variants found in homozygous regions, this usually reduces the number of variants by two orders of magnitude. These regions can also directly be analysed with our disease mutation identification tool MutationDistiller. The application comes with simple and intuitive graphical interfaces for data upload, analysis, and results. We kept the structure of HomozygosityMapper so that previous users will find it easy to switch. With AutozygosityMapper, we provide a fast web-based way to identify disease mutations in consanguineous families. AutozygosityMapper is freely available at https://www.genecascade.org/AutozygosityMapper/.

## Graphical Abstract

**Graphical Abstract:**
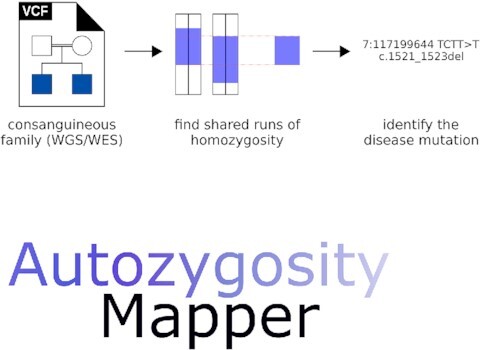
AutozygosityMapper: identify the disease mutation in deep-sequencing data from consanguineous families.

## INTRODUCTION

Homozygosity mapping is a common technique to study autosomal recessive disease in consanguineous families. The underlying assumption is that patients from consanguineous pedigrees are likely to have inherited the same disease allele in homozygous state from their ancestor; passed in heterozygous state from both parents. Whilst patients from different families are likely to have different disease haplotypes, patients from the same family should share the same ‘autozygous’ disease haplotype.

Homozygosity mapping can be performed with only the affected individuals, as was successfully shown in many studies, e.g. by Hildebrandt *et al.* (1). In this case, linkage analyses using SNP arrays or microsatellite markers will simply identify homozygous regions shared by the patients and there is no need to run a computationally intensive genome-wide linkage analysis. To facilitate such studies, we developed HomozygosityMapper (2), which searches for homozygous regions shared by all or many patients. In addition to much faster analyses, it is completely web-based and reduces the barriers for researchers and clinicians without bioinformatic expertise and without access to high-performance computing systems.

In the early 2010s, high-throughput sequencing became widely available and we released HomozygosityMapper2012 (3), which allowed the analysis of VCF files in addition to SNP array-based genotypes. However, in this release we treated the VCF genotypes as if they were SNP genotypes, i.e. they were stored as either homozygous for the reference allele, heterozygous, or homozygous for any other allele. While this method was successfully applied in many studies (e.g. (4–6)), it did not allow the export of potential disease variants for further downstream analysis.

With the shift from SNP arrays to high-throughput sequencing, today the exomes or genomes of single patients instead of whole pedigrees are analysed, leading to tens of thousands of variants if Whole Exome Sequencing (WES) is applied. While excluding common variants found in large-scale genotype repositories such as gnomAD (7) also leads to a drastic reduction, still several thousand variants remain in a typical WES project. Variants can subsequently be filtered for their effect on the protein with prediction tools, e.g. PolyPhen (8), SIFT (9), MutationTaster (10), CADD (11), or combinations of different tools. Searching for genotypes shared by all patients and not found in healthy relatives and the search for likely disease-causing genes with tools aimed at disease mutation identification such as eXtasy (12), the Exomiser (13), or MutationDistiller (14) lead to a further decrease. In patients from consanguineous families, the restriction to homozygous regions is another possible step in the identification of disease mutations.

Even though HomozygosityMapper2012 offered the option to download BED files with the coordinates of longer homozygous stretches found in all or many patients, the integration of this information into the WES analysis pipelines was cumbersome.

**Figure 1.**
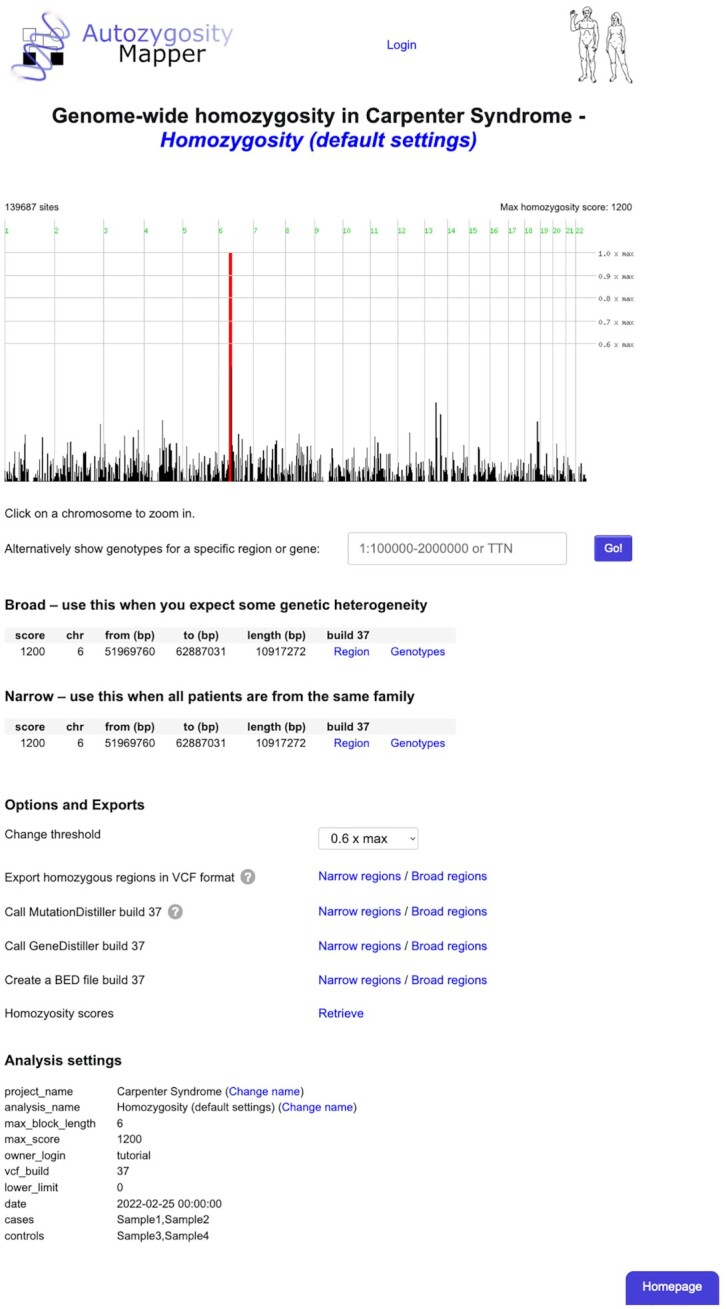
Genome-wide homozygosity. This figure depicts a genome-wide view of the runs of homozygosity found in the samples. The height of the peaks indicates the relative score of the shared homozygous segments, segments with a score higher than 60% of the maximum are shown in red. The genotypes are from the sample VCF file from our website.

We address this shortcoming with a novel release. AutozygosityMapper stores VCF genotypes as real genotypes, thereby allowing the search for potential autozygosity, i.e. regions in which the patients share exactly the same genotypes. Variants within homozygous or autozygous regions can be exported as VCF files for analysis with common disease mutation identification tools. In addition, we offer the possibility to directly transfer these variants to our tool MutationDistiller (14). As its predecessor, AutozygosityMapper can export BED files so that the detected regions (Figure 1) can also easily be studied in the IGV browser using the original BAM files.

**Figure 2.**
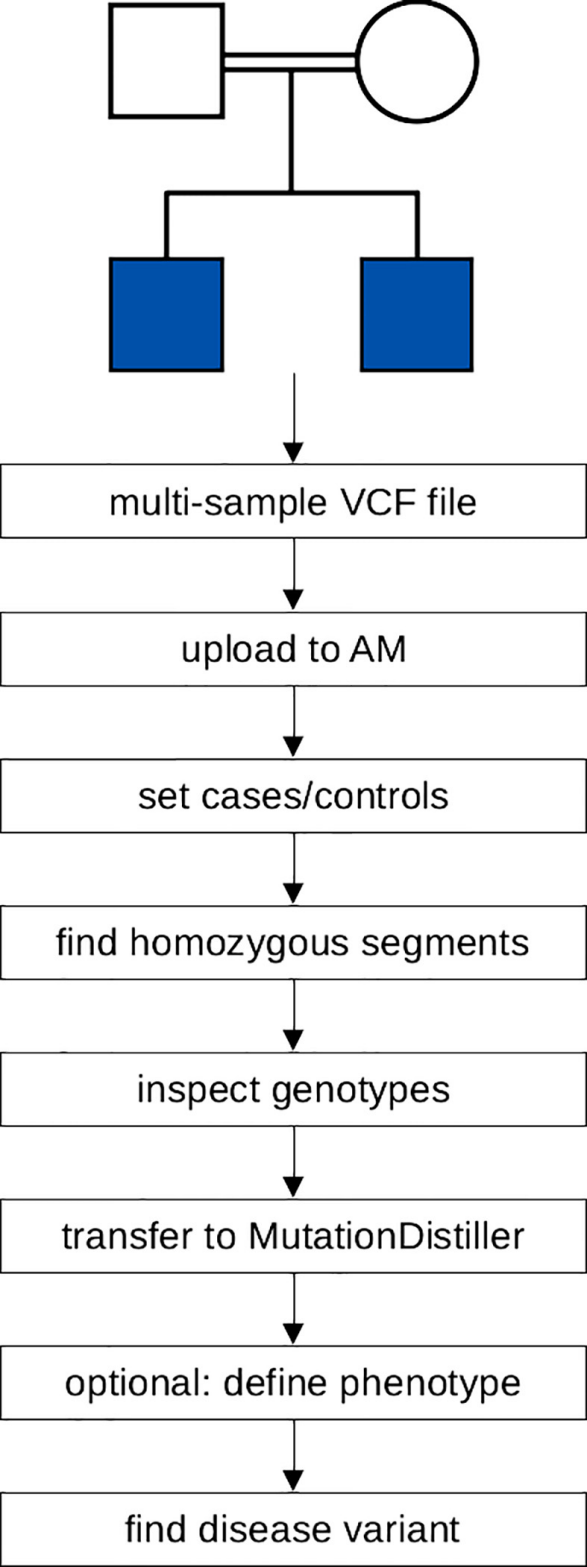
From VCF files to the disease mutation. This is a schematic depiction of AutozygosityMapper's analysis pipeline for VCF files. All steps from VCF file upload over the search for homozygous or autozygous regions to the search for the disease-causing variant with MutationDistiller are seamlessly integrated.

AutozygosityMapper differs from other tools aimed at the detection of runs of homozygosity or autozygosity such as PLINK (15) or AutoMap (16) because it does not offer any statistical measure of the degree of homozygosity. Instead, we provide a completely web-based pipeline that focusses on the visual inspection of the genotypes in the autozygous or homozygous regions and the direct identification of the disease mutation from multi-sample VCFs (Figure 2). The old version of HomozygosityMapper will remain in service and we recommend to use this tool for SNP array-based studies.

## CHANGES IN THE NEW VERSION

### Search for autozygosity

**Figure 3.**
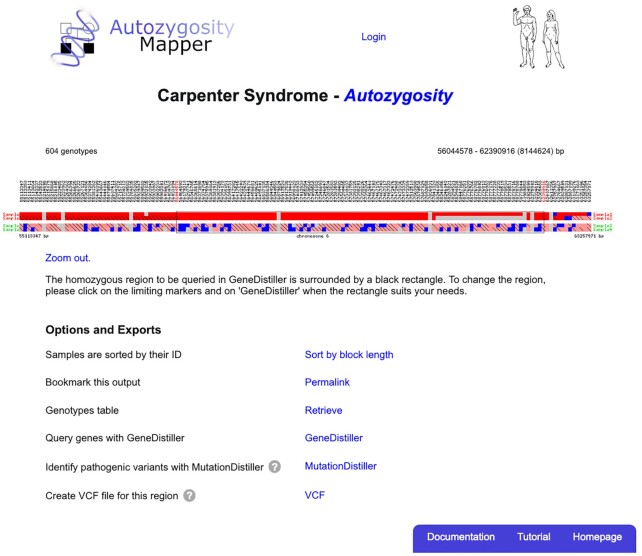
Genotypes in the autozygous region. Figure 3 shows the genotypes in the autozygous region shared by the two affected patients (Sample1, Sample2) with the other family members as controls shown below. Homozygous genotypes are indicated as red squares, a stronger colour indicates a greater length of the run of homozygosity in the sample. Heterozygous genotypes are shown in blue, no calls as grey boxes. Homozygous genotypes with different alleles carry a diagonal bar, indicating the end of the autozygous part of the shared homozygous region. The box around the autozygous segments depicts the border of the shared region as detected by AutozygosityMapper. These borders can be refined by mouse clicks if need be. The genotypes are from the sample VCF from our website.

**Figure 4.**
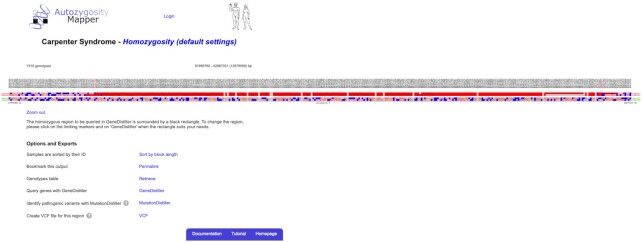
Genotypes in the homozygous region. This figure shows the homozygous region detected in the sample VCF from our website. Please note that this region is much longer than the autozygous region (depicted in Figure 3).

The old version converted the genotypes into 4 different states: (i) no call, (ii) homozygous for the reference allele, (iii) heterozygous, (iv) homozygous for any other allele. The new version stores single nucleotide variant (SNV) genotypes with their real alleles and can hence discriminate between the four different homozygous genotypes possible for a SNV. Using this information, the software can test whether different individuals are homozygous for the same haplotype, indicating identity-by-descent (IBD). While IBD is to be expected in members of the same family, the search for runs of homozygosity regardless of the haplotype is still possible to allow the search for disease-linked regions in patients from different families where different haplotypes are likely. Figures 3 and 4 illustrate the difference.

The genotypes of SNVs are stored in a bit-coded fashion to save disk space and gain speed. This is not possible for insertions and deletions (InDels) where a virtually unlimited number of alleles is possible. To increase the run-time performance, these are stored in a separate database table and not used in the mapping process. This also reduces possible errors due to sequencing and alignment artefacts which may occur in Illumina sequencing.

‘No call’ genotypes in SNP genotype files and VCF genotypes below the coverage threshold are treated as homozygous (but indicated as grey boxes in the genotype display). This allows to find long homozygous deletions in the patients, provided such information is included in the VCF file. Heterozygous deletions (or loss of heterozygosity) lead to hemizygosity, resulting in homozygous genotypes in the genotype file. Hemizygous regions are therefore discovered as if they were normal runs of homozygosity.

Users can decide whether they want to search for homozygous (i.e. all/many patients are homozygous for any allele) or autozygous regions (they share the same homozygous genotype); we recommend to use the latter only for patients from the same family.

In case of suspected locus heterogeneity, different analyses for the different families can be run without the need to re-upload the genotypes.

### Export of VCF files

The main application of WES/WGS is the identification of the variant causative for the observed phenotype. To limit the search to the variants found in homozygous or autozygous regions drastically reduces the number of variants to ‘positional candidates’.

AutozygosityMapper offers the export of VCF files containing only those variants. Users can decide whether they want to include all homozygous stretches or only the longest runs of homozygosity to be used. The VCF file contains all variants, including InDels and sites where different patients are homozygous for different alleles. The resulting VCF file can conveniently be uploaded to disease mutation search engines.

Because SNP arrays include ‘common’ polymorphisms but not rare disease variants, this option is not available for data from SNP chips.

### Search for causal variants with MutationDistiller

For human data, AutozygosityMapper offers the direct transfer of variants within homozygous or autozygous stretches to our disease mutation identification tool MutationDistiller, where deleteriousness predictions from MutationTaster (10) and phenotypic information can be used to further reduce the variants to those that are likely to disturb the function of candidate genes.

In addition, this enables the user to take advantage of the virtual gene panels from Genomic England PanelApp (17) to narrow the variants to those affecting known disease genes.

### Use of physical distance

HomozygosityMapper did not order runs of homozygosity by their physical length but instead by the number of homozygous markers. Many of our users requested to sort them by their real length instead, we have implemented this in the new release. To save the user from spurious results, e.g. only few homozygous genotypes flanking a long region not covered in the VCF file, AutozygosityMapper uses a threshold of at least 15 consecutive homozygous variants to treat a region as a run of homozygosity.

### Streamlined user interfaces

We overhauled the user interfaces to further facilitate the use of AutozygosityMapper. We kept the layout so that users of HomozygosityMapper can seamlessly use the new version, but streamlined the interfaces so that less clicks are required to study a project. This includes a clearer structure for the upload of genotype files and the display of the samples available in a project. The interface for sharing data with other registered users now uses autocompletion to facilitate the search for their account names.

The new version also offers a function to retrieve lost login credentials, which are automatically sent by email upon users' request. This is of course only possible for registered users, registration is however not mandatory to use AutozygosityMapper.

### Direct display of genomic regions or single genes

A frequent wish was the possibility to directly inspect the genotypes in a certain chromosomal region or around a candidate gene regardless of any run of homozygosity. This is now possible, users can either enter gene symbols or genomic regions they want to study in detail.

### Performance gains

The novel release of AutozygosityMapper is much faster than HomozygosityMapper. With our sample VCF data (WES with four samples), database import and identification of homozygous regions could be accomplished within 30 seconds instead of more than 60 seconds; the search for shared homozygous segments was completed within 6 seconds instead of 15 seconds.

A very large simulated WGS file with 17 million variants and 4 samples needed slightly longer than one hour for upload and identification of the homozygous regions (benchmarks are shown on the website).

### Email notification

Whilst upload and analysis of WES results or SNP array data for small families are usually accomplished in a few minutes, the analysis of larger projects with hundreds of samples takes longer. Registered users are now automatically informed by an email with a link to the results when the analysis has been completed.

### Mandatory use of https

While the old release worked without encryption, AutozygosityMapper requires SSL encryption (https) to ensure data privacy.

### Dropped features

We dropped several rarely used features and options of HomozygosityMapper to reduce the complexity and to speed up the analyses. A list of the changes can be found at https://www.genecascade.org/AutozygosityMapper/changes.html.

## DISCUSSION

AutozygosityMapper is, to our knowledge, the only web-based tool that supports multi-sample VCF files and offers the export of VCF files containing only variants that are ‘positional candidates’. The seamless integration of MutationDistiller provides a single pipeline for the identification of causal variants from VCF files in consanguineous families. We hope that AutozygosityMapper will empower users without bioinformatic expertise to include the search for runs of homozygosity or autozygosity into the search for disease mutations.

As its predecessor, AutozygosityMapper allows users to register themselves and use a login to upload, analyse, and share their data with other registered users. While it is not mandatory to use the software, we encourage all users to login when they work with patient data. This increases data security and allows the retrieval of results when access credentials are lost.

The homozygosity score given by AutozygosityMapper does not provide any measures of statistical significance for the runs of homozygosity or autozygosity it detects. Instead we provide capabilities to directly search for likely disease genes or even disease mutations contained in these stretches to reach the goal of gene hunting: The identification of the causal DNA variant.

### Use cases for AutozygosityMapper

The software is primarily intended to find disease-causing variants in WES/WGS data from patients from consanguineous human families. We recommend using the ‘autozygosity’ mode when a single family is studied as this will restrict the search to segments in which all patients share the same homozygous genotype. When different families are included, the ‘homozygosity’ mode should be used because this will search for regions which are homozygous in the patients but allow different haplotypes. If locus heterogeneity is expected (or there is no common homozygous region in the patients from different families), users should perform one analysis per family.

The software also works with single patients. Healthy family members or other controls are not needed to determine runs of homozygosity.

### Limitations

Whilst AutozygosityMapper is capable to find homozygous regions in species other than human, the main purpose of this release was the identification of disease mutations in humans and the options for other species are still limited.

AutozygosityMapper neither provides any statistical measures of homozygosity nor LOD scores. Should users wish to obtain such statistics, we recommend to use applications such as PLINK (15), AutoMap (16) or linkage analysis tools.

We provide only marginal support for SNP arrays since they contain common polymorphisms and there is no point in generating VCF files that do not include the disease mutation. For analysing SNP arrays, we recommend using HomozygosityMapper.

## OUTLOOK

### Genome version GRCh38

We are currently working on making genome build GRCh38 available in all our applications, including AutozygosityMapper and HomozygosityMapper.

### Search for homozygous deletions

A planned feature for the future is the explicit search for variants without genotypes in the affected individuals to easily identify homozygous deletions.

### Parallelisation

While the analysis of WES projects with less than 10 individuals is usually accomplished within minutes, Whole Genome Sequencing (WGS) projects with hundreds of samples need considerably more time. So far, such cases had been exceptional but they might become more frequent in the future due to the falling prices for WGS. Should the need arise, we will use a job scheduling system for the highly parallel analysis of genotypes.

## DATA AVAILABILITY

AutozygosityMapper is freely available at https://www.genecascade.org/AutozygosityMapper/. We provide sample data, a walk-through tutorial, concise documentation, a list of changes, and Frequently Asked Questions.

As the upload of genotypes is not possible for some users due to data protection rules, we also offer a download of our code. Unfortunately, AutozygosityMapper also needs a large database, specifically adapted to the server, to run reasonably fast so the source code alone is not enough to set up on-premises clones. The source code of AutozygosityMapper can be obtained from https://git-ext.charite.de/genecascade/autozygositymapper but we can offer only limited support.
